# Inclusion of people with disabilities in Chilean health policy: a policy analysis

**DOI:** 10.1186/s12939-024-02259-4

**Published:** 2024-08-29

**Authors:** Danae Rodríguez Gatta, Pamela Gutiérrez Monclus, Jane Wilbur, Johanna Hanefeld, Lena Morgon Banks, Hannah Kuper

**Affiliations:** 1 International Centre for Evidence in Disability, Department of Population Health, Faculty of Epidemiology and Population Health, London School of Hygiene and Tropical Medicine, London, WC1E 7HT UK; 2 Millennium Nucleus Studies on Disability and Citizenship (DISCA), Santiago, Chile; 3 Department of Occupational Therapy and Occupational Science, Faculty of Medicine, University of Chile, Santiago, Chile; 4 Centre for International Health Protection, Robert Koch Institute, Berlin, Germany; 5 Department of Global Health and Development, Faculty of Public Health and Policy, London School of Hygiene and Tropical Medicine, London, UK

**Keywords:** Health policy analysis, Health equity, People with disabilities, Chile, Disability-inclusive health, EquiFrame, Health policy triangle

## Abstract

**Background:**

Around 18% of the population in Chile has disabilities. Evidence shows that this population has greater healthcare needs, yet they face barriers to accessing healthcare due to health system failures. This paper aims to assess the inclusion of people with disabilities in health policy documents and to explore the perceptions of key national stakeholders regarding the policy context, policy processes, and actors involved.

**Methods:**

A policy content analysis was conducted of 12 health policy documents using the EquiFrame framework, adapted to assess disability inclusion. Documents were reviewed and rated on their quality of commitment against 21 core concepts of human rights in the framework. Key national stakeholders (*n* = 15) were interviewed, and data were thematically analysed under the Walt and Gilson Policy Analysis Triangle, using NVivo R1.

**Results:**

Core human rights concepts of disability were mentioned at least once in nearly all health policy documents (92%). However, 50% had poor policy commitments for disability. Across policies, *Prevention* of health conditions was the main human rights concept reflected, while *Privacy* of information was the least referenced concept. Participants described a fragmented disability movement and health policy, related to a dominant biomedical model of disability. It appeared that disability was not prioritized in the health policy agenda, due to ineffective mainstreaming of disability by the Government and the limited influence and engagement of civil society in policy processes. Moreover, the limited existing policy framework on disability inclusion is not being implemented effectively. This implementation gap was attributed to lack of financing, leadership, and human resources, coupled with low monitoring of disability inclusion.

**Conclusions:**

Improvements are needed in both the development and implementation of disability-inclusive health policies in Chile, to support the achievement of the right to healthcare for people with disabilities and ensuring that the health system truly “leaves no one behind”.

**Supplementary Information:**

The online version contains supplementary material available at 10.1186/s12939-024-02259-4.

## Abstract in Spanish

**Introducción:**

Alrededor del 18% de la población de Chile tiene discapacidad. Los datos demuestran que esta población tiene mayores necesidades de salud, pero se enfrenta a barreras para acceder a la salud debido a las deficiencias del sistema sanitario. El objetivo de este estudio es evaluar la inclusión de las personas con discapacidad en las políticas sanitarias y explorar las percepciones de actores nacionales en relación al contexto político, los procesos políticos y los actores implicados.

**Métodos:**

Se realizó un análisis de contenido de 12 políticas sanitarias utilizando el marco EquiFrame, adaptado para discapacidad. Se calificó la calidad de compromiso de las políticas con respecto a 21 conceptos de derechos humanos del EquiFrame. Se entrevistó a 15 actores nacionales, y los datos se analizaron temáticamente según el Triángulo de Políticas de Walt y Gilson, utilizando NVivo R1.

**Resultados:**

Los conceptos de derechos humanos en materia de discapacidad se mencionaron al menos una vez en casi todas las políticas sanitarias (92%). Sin embargo, en el 50% de los casos los compromisos políticos en materia de discapacidad eran escasos. En todas las políticas, la *Prevención* de los problemas de salud fue el principal concepto de derechos humanos reflejado, mientras que la *Privacidad* de la información fue el concepto menos mencionado. Los participantes describieron un movimiento de la discapacidad y una política sanitaria fragmentados, relacionados con un modelo biomédico dominante de la discapacidad. Pareciera que la discapacidad no es prioritaria en la agenda política sanitaria, debido a su ineficaz integración por parte del Gobierno y a la limitada participación de la sociedad civil en los procesos políticos. Además, el limitado marco político existente sobre salud inclusiva no se está implementando eficazmente. Esta deficiencia en la implementación se atribuyó a la falta de financiamiento, liderazgo y recursos humanos, junto con el escaso monitoreo de la discapacidad.

**Conclusiones:**

Se requieren mejoras tanto en el desarrollo como en la implementación de políticas de salud inclusivas de la discapacidad en Chile, para apoyar el alcance del derecho a la salud de las personas con discapacidad y asegurar que el sistema de salud realmente “no deje a nadie atrás”.

## Background

People with disabilities represent about 1.3 billion of the world’s population [1] and include *“those who have long-term physical*,* mental*,* intellectual or sensory impairments which in interaction with various barriers may hinder their full and effective participation in society on an equal basis with others”* [2]. On average, people with disabilities die 14 years earlier, including due to preventable health inequities [1, 3]. In Latin America and the Caribbean (LAC) there are about 85 million people with disabilities (15% of the total population) [4]. Systematic review evidence indicates that they use health services more frequently than the general population and face inequities in terms of coverage, quality, and affordability of healthcare, related to access barriers [5]. A meta-synthesis of qualitative studies conducted in LAC confirms that adults with disabilities face broad-ranging access barriers both in the supply and demand sides of primary healthcare (e.g., poor health worker training and inaccessible information) [6]. This overall situation in LAC is also apparent in Chile, the focus of the current study, where there are about 3 million people with disabilities (18% of the population) [7]. For instance, evidence from Chile shows that people with disabilities are more likely to experience difficulties accessing health centers [8] and are less likely to undergo cancer screening [9, 10]. Moreover, about 77% of primary healthcare workers report a lack of protocols for patients with disabilities, and 59% describe inaccessible health infrastructure [11].

In this context, it is crucial that people with disabilities are meaningfully involved and that their rights are promoted and mainstreamed in the health sector [1, 12]. Hence, to realize disability-inclusive care, health systems should “expect, accept, and connect” people with disabilities to access quality health services that are intentionally designed to include them, on an equal basis as those without disabilities and without incurring additional costs [13, 14]. The Missing Billion Framework identifies core components relevant for achieving disability-inclusive health, operating at the systems level (governance, leadership, financing, data and evidence), demand-side service level (autonomy and awareness, affordability) and supply-side service level (human resources, health facilities, rehabilitation and assistive technology) [14]. Achievement of disability inclusion across these components should improve health outputs for people with disabilities (e.g. service coverage) and therefore reduce inequities in health outcomes (e.g. mortality gaps) [14].

Barriers to accessing healthcare often arise from health system level failures, in particular lack of governance, as health policies are structural determinants of the organization of healthcare and health equity [15]. The policy framework in Chile appears to support the inclusion of people with disabilities in healthcare. In 2008, Chile ratified the United Nations Convention on the Rights of Persons with Disabilities (UNCRPD) [16] and in 2010 the National Disability Law was enacted [17]. Furthermore, the last National Health Strategy (2010–2020) included provisions for better access to rehabilitation, education of caregivers, and access to treatment for people with psychosocial disabilities [18]. However, amidst the upcoming review of Chile before the Committee on the Rights of Persons with Disabilities, civil society expressed concerns about implementation gaps in health [19]. For instance, they raised issues about the continuation of forced sterilization of women with disabilities and lack of health worker protocols for attending to patients with disabilities, inaccessible health information, and lack of mental health funding [19]. Moreover, there is evidence that the needs of people with disabilities were not fully addressed in government responses to COVID-19 in South America, including in Chile, where this group remained invisible in data collection for decision-making in public policy [20, 21].

Health policy analyses on disability are critical for understanding the gaps between policy formulation and implementation, the strengths and weaknesses of policy documents, and the level of commitment to disability [22–24]. However, health policies have been largely understudied using a disability lens. Previous policy analyses in Chile have focused on disability-specific policies [25, 26] or programs [27, 28]. Therefore, it is necessary to understand the broad health policy framework impacting access of people with disabilities to general healthcare across the Chilean health system. The aim of this study is to assess the inclusion of people with disabilities in Chilean general healthcare policy documents and to explore the perceptions of key national stakeholders regarding the policy context, policy processes, and actors involved.

## Methods

### Study design and setting

This study consisted of a policy content analysis of 12 policy documents and 15 key informant interviews. The study was conducted in Chile, a geographically diverse high-income country of 20 million inhabitants [29–31]. Chile has a two-tiered health system including both public and private insurance schemes and a mixed health service provision [32, 33]. It is led by the Ministry of Health, structured through the under secretariats of Public Health and Healthcare Networks. The National Health System of Healthcare Services includes 29 autonomous health services across 16 regions, overseeing mainly hospitals. Local municipalities manage the provision of decentralized primary healthcare services.

### Policy analysis

#### Selection of policies

Health policy documents were selected that fulfilled the following eligibility criteria: (1) overarching documents (policies, strategies, or plans), (2) issued by official government bodies (e.g. Ministry of Health (MoH)), (3) currently in force (i.e., published within the last 5 to 10 years, or targets not outdated), (4) of national scope, and (5) considered to relate to access to general healthcare for the overall population. Laws, technical guidance and recommendations were excluded. Key stakeholders related to disability policy and health systems in Chile were consulted to refine the selection criteria, including the MoH, the Ministry of Social Development and Family, the Pan American Health Organization and four academic experts. Eligible policies were searched through official websites of the national libraries of the MoH, the Ministry of Social Development and Family, and the National Congress of Chile.

#### Data extraction and analysis

The EquiFrame framework was used to guide the content analysis of health policy documents [34]. The EquiFrame is a systematic policy analysis framework developed to assess the inclusion of 21 core concepts of human rights and 12 vulnerable groups in health policies, to improve equity in healthcare. Each core concept has a description of its key language and questions, which were adapted to be relevant to people with disabilities and general healthcare (Table 1). For example, the key language for the concept of *Non-discrimination* was: *“Persons with disabilities are not discriminated against based on their distinguishing characteristics”*, and the key question: *“Does the policy support the rights of persons with disabilities with equal opportunity to receive healthcare?”*. Moreover, we searched for the explicit mention of “people with disabilities” within documents and what was defined as disability under each policy.

**Table 1:** List of EquiFrame adapted core concepts of human rights for people with disabilities

Nº	Concept	Key question	Key language
1	Non-Discrimination	Does the policy support the rights of persons with disabilities with equal opportunity to receive healthcare?	Persons with disabilities are not discriminated against on the basis of their distinguishing characteristics.
2	Individualized Services	Does the policy support the rights of people with disabilities with individually tailored services to meet their needs and choices?	People with disabilities receive appropriate, effective, and understandable services.
3	Entitlement	Does the policy indicate how people with disabilities may qualify for specific benefits relevant to them?	People with disabilities who have limited resources are entitled to some services free of charge or may be entitled to respite grant.
4	Capability-based services	Does the policy recognize the capabilities existing within people with disabilities?	For instance, peer to peer support among people with disabilities, advocacy groups and organizations of people with disabilities.
5	Participation	Does the policy support the right of people with disabilities to participate in the decisions that affect their lives and enhance their empowerment?	People with disabilities can exercise choices and influence decisions affecting their life. Such consultation may include planning, development, implementation, and evaluation.
6	Coordination of Services	Does the policy support assistance of people with disabilities in accessing services from within a single provider system (intragency) or more than one provider system (inter-agency) or more than one sector (inter- sectoral)?	People with disabilities know how services should interact where inter-agency, intra- agency, and inter-sectoral collaboration is required.
7	Protection from harm	Are people with disabilities protected from harm during their interaction with health and related systems?	People with disabilities are protected from harm during their interaction with health and related systems.
8	Liberty	Does the policy support the right of people with disabilities to be free from unwarranted physical or other confinement?	People with disabilities are protected from unwarranted physical or other confinement while in the custody of the service system/provider.
9	Autonomy	Does the policy support the right of people with disabilities to consent, refuse to consent, withdraw consent, or otherwise control or exercise choice or control over what happens to him or her?	People with disabilities can express “independence” or “self-determination”. For instance, person with an intellectual disability will have recourse to an independent third-party regarding issues of consent and choice.
10	Privacy	Does the policy address the need for information regarding people with disabilities to be kept private and confidential?	Information regarding people with disabilities need not be shared among others.
11	Integration	Does the policy promote the use of mainstream services by people with disabilities?	People with disabilities are not barred from participation in services that are provided for general population.
12	Contribution	Does the policy recognize that people with disabilities can be productive contributors to society?	People with disabilities make a meaningful contribution to society.
13	Family Resource	Does the policy recognize the value of the family members of people with disabilities in addressing health needs?	The policy recognizes the value of family members of people with disabilities as a resource for addressing health needs.
14	Family Support	Does the policy recognize individual members of people with disabilities may have an impact on the family members requiring additional support from healthcare services?	Caring for persons with disabilities may have mental health effects on other family members, such that these family members themselves require support.
15	Cultural responsiveness	Does the policy ensure that services respond to the beliefs, values, gender, interpersonal styles, attitudes, cultural, ethnic, or linguistic, aspects of the person?	i) People with disabilities are consulted on the acceptability of the service provided. ii) Health facilities, goods and services must be respectful of ethical principles and culturally appropriate, i.e. respectful of the culture of people with disabilities
16	Accountability	Does the policy specify to whom, and for what, services providers are accountable?	People with disabilities have access to internal and independent professional evaluation or procedural safe guard.
17	Prevention	Does the policy support people with disabilities in seeking primary, secondary, and tertiary prevention of health conditions?	
18	Capacity building	Does the policy support the capacity building of health workers and of the system that they work in addressing health needs of people with disabilities?	
19	Access	Does the policy support people with disabilities – physical, economic, and information access to healthcare services?	People with disabilities have accessible health facilities (i.e., transportation; physical structure of the facilities; affordability and understandable information in appropriate format).
20	Quality	Does the policy support quality services to people with disabilities through highlighting the need for evidence-based and professionally skilled practice?	People with disabilities are assured of the quality of the clinically appropriate services.
21	Efficiency	Does the policy support efficiency by providing a structured way of matching health system resources with service demands in addressing health needs of people with disabilities?	

*Note* Concepts adapted from Amin M, MacLachlan M, Mannan H, El Tayeb S, El Khatim A, Swartz L, et al. EquiFrame: a framework for analysis of the inclusion of human rights and vulnerable groups in health policies. Health Hum Rights. 2011;13:1–20, and Wilbur J, Scherer N, Mactaggart I, Shrestha G, Mahon T, Torondel B, et al. Are Nepal’s water, sanitation and hygiene and menstrual hygiene policies and supporting documents inclusive of disability? A policy analysis. Int J Equity Health. 2021;20:157

Core concepts were then translated into Spanish and the translation was checked by an external assessor (Additional File 1). Support was sought from the authors of the EquiFrame to review and approve the adaptations. Two reviewers (DRG and PGM) independently assessed each policy document for the inclusion of core concepts. Referenced concepts were rated based on their quality of commitment on a continuum from 1 (i.e., only mentioned) to 4 (i.e., intention to monitor) and then extracted and recorded in a Microsoft Excel spreadsheet (Table 2).

**Table 2:** Scoring of quality of commitment and summary indices

Scoring	Quality of commitment
0	Concept not mentioned
1	Concept only mentioned
2	Concept mentioned and explained
3	Specific policy actions identified to address the concept
4	Intention to monitor concept was expressed
**Summary indices**	
Each policy	**Core concept coverage [(n/21) x 100]**: the proportion (%) of core concepts included in a policy, where n is the number of core concepts rated above 0 and 21 is the total number of core concepts.
	**Core concept quality [(n/N) x 100]**: the proportion (%) of core concepts included in a policy with top quality, where n is the total number of core concepts rated “3” or “4” and N is the total number of core concepts referenced. *
Across policies	**Total references [(n/377) x 100]**: the proportion (%) of references to core concepts across policies, where n is the total number of references made to a core concept and 377 is the total number of references to all core concepts across policies.
	**Average score [(n/N)]**: the average score of core concepts across policies, where n is the total number of references to a core concept and N is the total score of the concept across all policies

* Several references to a single core concept can be found in each policy

The scores were compared and aligned by the reviewers, after resolving any discrepancies. Four summary indices were developed: core concept coverage, core concept quality, core concept reference, and average score (Table 2).

### Key informant interviews

#### Sampling and recruitment

Fifteen key national stakeholders were interviewed to explore the policy context, process and actors involved (Table 3). A stratified purposive sampling was applied to ensure the representation of different views and expertise of sectors related to health policy and disability. We recruited participants through recommendations of governmental officials and academic experts. Potential participants were also identified from policy documents. For example, authors, contributors, technical advisors, and those designated to implement and monitor policies.

**Table 3:** Participants of key informant interviews (n = 15)

Sector	Department, Institution
Government (n = 6)	1) Rehabilitation and Disability, Ministry of Health
	2) Mental Health, Ministry of Health
	3) Non-Communicable Diseases, Ministry of Health
	4) Care Network Management, Ministry of Health
	5) Autonomy and Dependency, National Disability Agency
	6) Evaluation and Studies, National Disability Agency
Parliament (n = 2)	7) Senate, National Congress of Chile
	8) Chamber of Deputies, National Congress of Chile
Health provider (n = 3)	9) National Specialized Referral Hospital
	10) Life Cycle, Regional Health Service
	11) Life Cycle, Regional Health Service
Civil Society (n = 2)	12) Human Rights Organization
	13) Patients’ Association
International (n = 2)	14) Mental Health, Pan American Health Organization
	15) Special Envoy, United Nations Secretary-General

*Note* The National Disability Agency (in Spanish, *Servicio Nacional de la Discapacidad*) is under the Ministry of Social Development and Family

#### Data collection and analysis

Interviews were held in Spanish between October and December 2022. Most interviews were conducted face-to-face at the participant’s workplace or public locations, although some were online through Zoom, due to COVID-19 pandemic public health regulations. Semi-structured interview guides with open-ended questions were used to frame discussions with participants. Interviews lasted between 45 and 60 min and were audio-recorded and transcribed. The Walt and Gilson Policy Analysis Triangle was used to guide the analysis of the key informant interviews [35, 36]. This framework presents a simplified model of the complex interplay between health policy content, systemic factors of the policy context, policy making processes, and the actors involved in a particular issue [35, 36]. Interview transcripts were analysed thematically supported by the NVivo R1 software. Audio recordings were transcribed in Spanish and only selected quotes were translated into English; the quality of the translation was assessed by an external assessor. Transcriptions were coded deductively, with preliminary codes developed based on the interview guide and the emergent topics of the interview. Codes were selected based on frequency, relevance to the research question and level of divergence, and final themes were developed. Codes were revised by co-authors and triangulated with the health policy documents to corroborate information. This study received ethical approval from the Ethics Committees of University of Chile and London School of Hygiene and Tropical Medicine.

## Results

### Summary indices of the policy content analysis using the EquiFrame

**Fig. 1:**
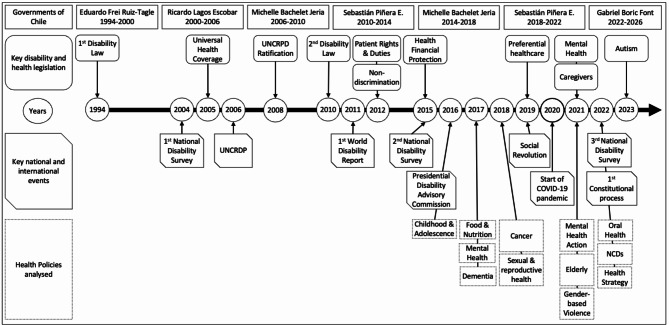
Timeline of key legislation, events, and health policies analysed. *Legend* UNCRPD, United Nations Convention on the Rights of People with Disabilities; NCDs, Non-Communicable Diseases

Twelve policy documents were analysed (*n* = 1 (8%) strategy, *n* = 4 (33%) policies, and *n* = 7 (58%) plans) (Table 4, Additional File 2, and Fig. 1) [35–46]. Core human rights concepts of people with disabilities were referenced, at least once, in nearly all policies (*n* = 11, 92%), except for the National Food and Nutrition Policy. The National Mental Health Plan had the highest reference to core concepts (90%), followed by the National Health Strategy (76%) (Table 4). In contrast, the National Plan on Cancer (5%) and Non-Communicable Diseases (10%) had few references to core concepts. However, the high number of references did not reflect the highest strength of policy commitment. For instance, only 1% of concepts referenced in the National Health Strategy described specific policy actions or monitoring of interventions for people with disabilities. The highest quality of commitment was found in the National Mental Health Action Plan (91%), followed by the National Oral Health Plan (83%). Overall, 50% of policies had low (0–3%) quality of commitment.

**Table 4:** Core concept coverage and quality of health policy documents included (n = 12)

Year	Title	Core concept coverage (%)	Core concept quality (%)*
2016	National Policy on Childhood and Adolescence	48%	12%
2017	National Plan on Dementia	62%	0%
2017	National Plan on Mental Health	90%	28%
2017	National Policy on Food and Nutrition	0%	0%
2018	National Policy on Sexual and Reproductive Health	38%	0%
2018	National Plan on Cancer	5%	33%
2021	National Health Policy to address Gender-Based Violence	52%	3%
2021	National Health Plan for the Elderly and its Action Plan	38%	38%
2021	National Action Plan on Mental Health	38%	91%
2022	National Health Strategy for the 2030 Health Goals	76%	1%
2022	National Plan on Non-Communicable Diseases	10%	0%
2022	National Plan on Oral Health	14%	83%

*Core concepts scored 3 (specific policy actions identified to address the concept) or 4 (intention to monitor concept was expressed)

Table 5 shows the aggregated results across policy documents. The concepts of *Prevention* (17%), *Entitlement* (12%) and *Individualized services* (11%) were the top three most frequently mentioned concepts across all policies analysed. The least referenced concept was *Privacy* (0.3%), although several others were also infrequently mentioned (1%) including *Capability-based services*, *Contribution*, *Cultural responsiveness*, and *Efficiency*. The highest average quality scores were found in *Capacity Building* (2.7), and *Coordination of Services* (2.2). Again, frequency and quality could differ, as with *Accountability*, which only represented 1% of total references but obtained the highest average score of 3.5. Examples of references scored 3 or 4 are in Additional File 3.

**Table 5:** References to core concepts and average score across health policy documents (*n* = 12)

Nr.	Concept	Health policies (n = 377 references across policies)	
		Total references (%)	Average quality score (max. 4)
1	Non-Discrimination	8%	1.4
2	Individualized Services	11%	1.9
3	Entitlement	12%	2.0
4	Capability-based services	1%	1.5
5	Participation	3%	1.6
6	Coordination of Services	8%	2.2
7	Protection from Harm	7%	1.8
8	Liberty	2%	1.6
9	Autonomy	5%	1.7
10	Privacy	0.3%	1.0
11	Integration	4%	1.9
12	Contribution	1%	1.5
13	Family Resource	2%	1.0
14	Family Support	2%	1.3
15	Cultural responsiveness	1%	1.5
16	Accountability	1%	3.5
17	Prevention	17%	1.6
18	Capacity building	2%	2.7
19	Access	7%	1.8
20	Quality	6%	1.7
21	Efficiency	1%	2.0
	Total	100%	

### Key informant interviews

#### A fragmented disability movement and health policy

A key issue raised by participants was that fragmentation in the disability movement weakens their influence on the health policy agenda. Interviewees reported that civil society organizations were grouped by impairments or medical diagnoses and advocated for their own health needs, rather than for disability inclusion more holistically.

Fragmentation was also reflected in health policies, which were described as hyper-focalized by health conditions, instead of being formulated more comprehensively for all disabilities. In addition, it was argued that the focus has been on physical and sensory impairments rather than intellectual and psychosocial impairments. This was viewed as inefficient for policy processes. Some participants considered that inclusive health has received some government attention, but mostly focusing on people with autism, thus reinforcing fragmentation.

Participants expressed concerns about parliamentarians supporting causes advocated for by civil society, regardless of rational health prioritizations. They suggested that policy makers should lead policy formulation focusing holistically on the needs of people with disabilities. Ideally, solutions would include a comprehensive and intersectoral disability-inclusive health policy, and mainstreaming of disability in existing health policies.

#### Role of international agencies in disability perspectives

Two international initiatives were perceived as key to shaping the disability landscape in Chile, by introducing elements of a more social perspective of disability. The World Health Organization (WHO), well recognized as an influential governing body, installed the International Classification of Functioning, Disability and Health (ICF) and biopsychosocial model of disability in Chile. Furthermore, the UNCRPD installed a human rights perspective and was recognized as a relevant legal framework for policy. Nevertheless, some participants reported that the dominant model of disability in Chile remains biomedical.

*“Unfortunately*,* people with disabilities in the health movement are not considered*,* unless they belong to an organization that obeys a pathologizing or biomedical model […] But these organizations obey the past model*,* the past! They relate to health in a charitable*,* rehabilitative way*,* not in a model of inclusion.”* Interview 2, civil society organization.

#### Disability as low politics in the health policy agenda

Inclusion of all people with disabilities in general healthcare was viewed as a low priority issue for regional or central government. For instance, government officials noted that the accessibility of health services for people with disabilities has remained as a government measure, but without an implementation strategy. Moreover, participants especially argued that there is a lack of policy actions for people with disabilities in the Sexual and Reproductive Health (SRH) Policy, as reaffirmed in this content analysis. Improvement of disability awareness and accessibility mindset among policy makers from the start of policy formulation was seen as fundamental for improving the prioritization of disability, especially in the Ministry of Health.

#### Ineffective mainstreaming of disability and coordinated action among governing bodies

Government officials consistently noted that disability-related policy is mainly led by the Rehabilitation and Disability Department of the MoH. The Department has an acknowledged role in ensuring disability is mainstreamed across teams, but was criticized for a low interdepartmental work by government officials. However, it was also agreed that it is a general Ministerial challenge to integrate actions across sub-secretaries of health.

Moreover, disability was described as a cross-cutting issue, which should be addressed by several ministries and a complex network of actors, besides the health sector alone. However, government officials and civil society actors noted a lack of coordinated action between ministries. There is a relevant agency - National Disability Agency (NDA; *Servicio Nacional de la Discapacidad*,* SENADIS*, for its Spanish abbreviation) of the Ministry of Social Development and Family – whose formal role is to lead disability inclusion in the policy agenda of all governing bodies. However, their effectiveness in fulfilling this role was questioned by participants.

*“The NDA is not an institution that is truly a governing body*,* in terms of putting the issue of disability and inclusion as strongly as it needs to be.”* Interview 7, ex-member of parliament.

#### Civil society’s limited influence in the health policy agenda and engagement in policy formulation

Participants identified four main pathways for influencing the health agenda by civil society, including Organizations of People with Disabilities (OPDs). The first is through advocacy to parliament. The second route includes the participation of people with disabilities in temporary task forces led by the Executive, where issues are recognized. However, civil society and international actors reported that even though task forces were created, policy implementation was uncertain. Third, it is possible for people with disabilities to raise issues at primary care level, when health teams lead participatory assessments of the population’s health needs. Finally, the judicialization of cases and the media has been used by civil society to increase pressure and promote disability inclusion.

However, government officials perceived that OPDs have not managed to influence the health agenda and noted that they still lack capacity for health policy making. A government official also reported that many organizations of civil society lack a structure, are difficult to reach or are not interested in participating in policy processes. One exception highlighted was the autism movement.

*“We are recognizing that organizations of civil society are valid actors for needs assessments. This is how is it being done*,* so when they work on autism*,* all [autism] organizations participate in the parliament.”* Interview 5, government official.

Furthermore, participants reported a limited engagement of civil society in health policy formulation. Their participation is not institutionalized in the MoH, and it depends on the political will of policy makers. Government officials considered that OPDs should be involved in policy design, although their inclusion has been slow. The institutional culture of Government was reported to have acted as a barrier, as technical expert knowledge is prioritized over lived experience of disability.

#### Gap in implementation of the existing limited policy framework for disability inclusion

Some disability inclusion was reported in the National Health Strategy and the national policies on Mental Health, Elderly, and Childhood and Adolescence, which supports the findings of the document review. The Preferential Care Law for Elderly and People with Disabilities was also regarded as a relevant policy framework for disability inclusion, as it guarantees priority access to appointments, emergencies, medicines, and examinations [37]. However, participants identified a gap between policy formulation and implementation. For example, government officials and health providers reported that the monitoring of the Preferential Care Law revealed a lack of execution, poor preparation of health teams and limited public information on this law.

*“The health centers were not implementing this [Preferential Care Law] […] The teams were not prepared. So*,* while it is true that the spirit of the law is fine*,* it is often the case that to apply these laws*,* some kind of resources are needed.”* Interview 12, health provider.

#### Lack of financing, leadership, and human resources affecting policy implementation

Participants identified three main reasons for the policy implementation gap. These included: lack of disability financing, an inconsistent political approach to disability and lack of leadership, and gaps in human resources.

Lack of resources for disability and lack of disability-related pay for performance indicators – the main incentive mechanism for primary care teams [33] – were identified by participants as barriers for policy implementation. For example, the SRH policy did not include resources to improve the accessibility of infrastructure.

*“But certainly*,* one of the problems that we have had in Chile and in many other countries is that action plans are not associated to budget*,* or budgets are so low that they do not relate to the objectives set for the action plan […] This causes many problems*,* because it is a dead public policy at the end*,* without any type of effect”.* Interview 15, international actor.

The policy implementation gap was also attributed to changes in government or political authorities and lack of leadership. The political will and perspectives of the government in power and the legislature were recognized as key factors in prioritizing health issues and successful policy implementation (specially from the President and mid-level policy officers). However, regardless of who is in power, the approach to disability appeared inconsistent as policies are either discontinued or restarted from scratch. Moreover, some participants perceived that there was no strong leadership on disability in central government, with diffused responsibilities between the MoH and the NDA. Similarly, at the regional level, health providers considered that disability leadership is fragmented.

*“In a way*,* I am in charge of disability*,* I see the whole musculoskeletal*,* neurological rehabilitation program […] It is kind of fragmented*,* there is no unit that concentrates a strategic and cross-cutting view of disability”* Interview 12, health provider.

Different policy solutions were identified to improve leadership on disability inclusion. Government officials and health providers suggested the implementation of dedicated disability units at central and regional levels, while members of parliament proposed a new interministerial governance. Other participants proposed strengthening existing leadership or reinforcing interdepartmental and multisectoral work.

Finally, implementation is perceived to be affected by gaps in human resources. Participants identified a lack of personnel to implement additional services, lack of training among health teams about disability, and high health professional turnover.

#### Low monitoring of disability inclusion

Government officials, health providers, and civil society actors observed limited monitoring of disability inclusion in general healthcare policies. For example, they reported that the current National Health Strategy includes indicators on health conditions but not on disability. Government officials, health providers, and members of parliament suggested that monitoring could be strengthened through official complaints or consultations with civil society. Finally, international and civil society actors highlighted the need for an independent disability monitoring mechanism in Chile.

## Discussion

This health policy analysis on disability in Chile included a content analysis of 12 policy documents on general healthcare and 15 key informant interviews. Disability was mentioned in nearly all health policy documents reviewed (92%). However, 50% of policies had low or no policy commitments to disability. *Prevention* was the main human rights concept reflected across policies, while *Privacy* was the least referenced concept. Furthermore, interviews revealed a fragmented disability movement and health policy, related to a dominant biomedical model of disability. It appeared that disability was not prioritized in the health policy agenda due to ineffective mainstreaming of disability from Government and the limited influence and engagement of civil society in policy processes. Moreover, the limited existing policy framework on disability inclusion is not being implemented. Lack of financing, leadership, and human resources were attributed to this implementation gap, coupled with low monitoring of disability inclusion.

Most mainstream Chilean health policies analysed in this study included at least one core human rights concept of people with disabilities. In contrast, previous studies using the EquiFrame found more limited reference to people with disabilities in water, sanitation and hygiene (WASH) policies of Nepal, Bangladesh and Cambodia [22, 23]. Similarly, an international study on WASH policy during the COVID-19 pandemic found gaps in attention to disability [38]. Whilst reference to disability was higher in Chile, the low policy commitment to disability in Chile’s health policies was consistent with previous research on WASH reporting almost non-existent actions for disability [22]. Our findings indicated that policies mainly focused on the prevention of health conditions, although with a stronger emphasis on preventing disability rather than improving access to preventive healthcare among people with disabilities. In contrast, previous analyses highlighted a focus on infrastructure and information accessibility [22, 23, 38].

Our findings showed only an incipient inclusion of disability and human rights perspectives in health policy. This appears not to be exclusive to disability, however, but also more generally. A policy analysis of 171 documents found a lack of human-rights perspective in public policy in Chile [36]. In addition, previous studies in Chile analysing disability-specific policies and programs found varying levels of inclusion of health as a right of people with disabilities [25–28]. Nevertheless, our findings suggest a continuation of a biomedical framing of disability, which remains engrained in health systems [39]. Health policies were described as hyper-focalized by health conditions or impairment type. Similarly, the disability movement was depicted as fragmented by medical diagnoses. Their influence seemed to be limited to health needs assessments without further involvement in policy processes, even though their participation in policies that concern them is imperative [2, 4]. This has been pointed out as one of the weaknesses of the Chilean Health System, where there is a lack of engagement with other stakeholders of civil society in public health [31].

Our analyses suggested that there was some inclusion of disability in government discourse, but with poor policy implementation strategies and resources. This has been similarly observed in African Union Policies [40] and in the Philippines [41]. Thus, this raises questions about both the quality of health policies but also the actual prioritization of disability in Chile. An analysis of Chilean public policy with a human rights approach found that policy instruments were of low quality, as they lacked structure, budget, and mechanisms for monitoring, accountability and participation [42]. Additionally, disability was regarded as an issue of “low politics”. During the COVID-19 pandemic, Chile prioritized populations within national plans based on their clinical risk, whereas the health systems of the Bahamas, Mexico, and Peru prioritized populations given their vulnerability (e.g. people with disabilities and migrants) [43]. Despite Chile‘s prioritisation of people with certified and severe disabilities for COVID-19 vaccination [44], evidence suggests that the government lacked a comprehensive strategy to fully address the needs of all people with disabilities [20]. Furthermore, questions on disability were excluded from the pandemic version of a national survey, a key instrument used to identify and prioritise groups for public policy [21].

Poor policy commitment to disability in paper is compounded by the lack of policy execution in practice. This issue was well illustrated in the Sexual and Reproductive Health Policy [45], which indicated no strong commitment about disability and poor implementation, also due to a lack of resources. These policy and implementation gaps may help to explain why women with disabilities in Chile have lower coverage of cancer screening services [9, 10] and face critical gaps in SRH services [19, 46]. Challenges with implementation of disability-inclusive policies have been observed in other settings, such as in Uganda for SRH [47], and in relation to COVID-19 responses in South America [20].

Some limitations of this study should be noted. The lack of mainstreaming of disability and coordinated action was reflected in the poor harmonization of disability models and terminology across health policy documents. This issue was evident in the previous 2011–2020 National Health Strategy of Chile, whose disability indicators could not be monitored due to changes in the conceptualization of disability [48], and it has been an issue similarly observed for older people in Chile [49]. Therefore, as we only considered the explicit mention of people with disabilities, some references of groups that could have experienced disability could have been missed (e.g. children with special healthcare needs, elderly with dependency). Furthermore, health policies using universal terms (e.g., “all”, “entire population”, “everybody”) could have implicitly included people with disabilities. However, it has been recognized that not explicitly targeting minorities or vulnerable groups could further perpetuate health inequities [1, 34, 50].

Moreover, participants had different conceptions of health policy, and the type of documents they translated into, which has been previously found [42]. Thus, there was not a complete overlap between our document selection and what participants referred to as health policy. In addition, our identification of documents could have been subject to selection bias and some health policies may have been missed. We also acknowledge the relevance of other social determinants of health; however, these were beyond the scope of this study [15]. Future assessment could be enriched with the analysis of additional multisectoral policies (e.g. housing, transportation, etc.). Despite these limitations, the strengths of this study lie in using a structured tool that allowed a systematic and independent assessment of documents by two reviewers who were native Spanish speakers and familiar with the context. In addition, data could be triangulated with information from key informant interviews.

## Conclusion

Improvements are needed in both the development and implementation of disability-inclusive health policies in Chile, to support the achievement of the right to healthcare for people with disabilities, and ensuring that the health system truly “leaves no one behind”.

### Electronic supplementary material

Below is the link to the electronic supplementary material.

Supplementary Material 1

Supplementary Material 2

Supplementary Material 3

## Data Availability

The dataset generated during the current study is not publicly available due to the privacy of individuals that participated in the study but is available from the corresponding author on reasonable request.

## Declarations

### Reflexivity statement

Our team reflects a diverse group of women from high-income countries (Canada, Chile, Germany, and the United Kingdom) and different ethnic backgrounds (e.g. White, Latino). We represent a mixed positioning on disability, including disability allies and lived family experiences of disability. The team is multidisciplinary, and different sectors are represented (government, academia, disability, health), with experience in conducting analysis of policies using similar methods in other countries. Co-authors reflect diverse seniority levels in research, ranging from early career to senior researchers. These varied backgrounds ensured that we considered the complexity of disability, considering the different needs across types of disabilities, ages, and genders. This approach allowed us to evaluate health policies through a comprehensive and inclusive lens.

## Abbreviations

ICF: International Classification of Functioning, Disability and Health
LAC: Latin America and the Caribbean
MoH: Ministry of Health
NDA: National Disability Agency
OPDs: Organizations of Persons with Disabilities
SRH: Sexual and Reproductive Health
UNCPRD: United Nations Convention on the Rights of Persons with Disabilities
WASH: Water, Sanitation and Hygiene
