# Supplementary material for: Inclusion of people with disabilities in Chilean health policy: a policy analysis
Source: Int J Equity Health. 2024 Aug 29;23:174. doi: 10.1186/s12939-024-02259-4 (PMC11360718; doi:10.1186/s12939-024-02259-4)
Supplement: Supplementary file 1 — Supplementary Material 1 [file 12939_2024_2259_MOESM1_ESM.docx]

| **Additional File 1. List of EquiFrame concepts in Spanish** | | | |
| --- | --- | --- | --- |
| **Nº** | **Concepto Clave** | **Lenguaje Clave** | **Pregunta Clave** |
| 1 | No discriminación | Las personas con discapacidad no son discriminadas en base a sus características distintivas. | ¿La política apoya los derechos de las personas con discapacidad con igualdad de oportunidades para recibir atención en salud? |
| 2 | Servicios personalizados | Las personas con discapacidad reciben servicios apropiados, efectivos, y comprensibles. | ¿La política apoya los derechos de las personas con discapacidad con servicios personalizados para satisfacer sus necesidades y elecciones? |
| 3 | Derecho/Garantía | Las personas con discapacidad que tienen escasos recursos, tienen derecho a algunos servicios gratuitos o asignación monetaria de respiro para cuidadores. | ¿La política indica como las personas con discapacidad podrían calificar beneficios específicos y relevantes para ellas/os? |
| 4 | Servicios basados en competencias | Reconoce a las personas con discapacidad y sus agrupaciones como actor/es relevantes. Por ejemplo, grupos de apoyo de pares entre personas con discapacidad, grupos de abogacía, u organizaciones de personas con discapacidad. | ¿La política reconoce las competencias existentes en las personas o grupos de personas con discapacidad? |
| 5 | Participación | Las personas con discapacidad puede elegir e influenciar decisiones que afectan sus vidas. Esta consulta puede incluir la planificación, el desarrollo, la implementación, y la evaluación. | ¿La política apoya el derecho de personas con discapacidad a participar en las decisiones que afectan a sus vidas y a potenciar su empoderamiento? |
| 6 | Coordinación de servicios | Las personas con discapacidad saben cómo los servicios deben interactuar cuando se requiere una colaboración interinstitucional, intrainstitucional, e intersectorial. | ¿La política apoya la asistencia a personas con discapacidad para que accedan a los servicios desde un único sistema de prestación (intrainstitucional) o más de un sistema de prestación (interinstitucional) o más de un sector (intersectorial)? |
| 7 | Protección contra daños | Las personas con discapacidad están protegidas de daños durante su interacción con el sistema de salud y otros sistemas afines. | ¿Las personas con discapacidad están protegidas contra daños durante su interacción con el sistema de salud y otros afines? |
| 8 | Libertad | Las personas con discapacidad están protegidas contra el confinamiento físico o de otros tipos injustificado mientras están bajo la custodia del sistema/prestador de servicios. | ¿La política apoya el derecho de las personas con discapacidad a estar libres de confinamiento físico o de otro tipo, injustificado? |
| 9 | Autonomía | Las personas con discapacidad puede expresar su auto-determinación. Por ejemplo, una persona con discapacidad intelectual podrán recurrir a una tercera persona independiente en cuestiones de consentimiento y elección. | ¿La política apoya el derecho de personas con discapacidad a consentir, negar el consentimiento, retirar el consentimiento, o de algún modo controlar o elegir sobre lo que les sucede? |
| 10 | Privacidad | La información sobre las personas con discapacidad no debe compartirse con otras personas. | ¿La política aborda la necesidad de mantener la privacidad y confidencialidad de la información sobre las personas con discapacidad? |
| 11 | Integración | A las personas con discapacidad no se les impide participar en los servicios que se proveen a la población general. | ¿La política promueve el uso de los servicios generales por parte de las personas con discapacidad? |
| 12 | Contribución | Las personas con discapacidad hacen una contribución significativa a la sociedad. | ¿La política reconoce que las personas con discapacidad pueden contribuir de forma productiva a la sociedad? |
| 13 | Recurso familiar | La política reconoce el valor de los familiares de las personas con discapacidad como un recurso para abordar las necesidades de salud. | ¿La política reconoce el valor de los familiares de las personas con discapacidad en el abordaje de las necesidades de salud? |
| 14 | Apoyo familiar | El apoyo/cuidado hacia personas con discapacidad puede tener efectos en el bienestar de otros familiares, de manera que estos mismos familiares requieren apoyo. | ¿La política reconoce que las personas con discapacidad pueden tener un impacto en el bienestar de los familiares requiriendo apoyo adicional de los servicios sanitarios? |
| 15 | Sensibilidad cultural | i) Las personas con discapacidad son consultadas sobre la aceptabilidad del servicio entregado. ii) Los establecimientos, los bienes y los servicios deben ser respetuosos con los principios éticos y culturalmente adecuados, es decir, respetuosos con la cultura de las personas con discapacidad. | ¿La política garantiza que los servicios respondan a las creencias, valores, género, estilos interpersonales, actitudes, aspectos culturales, étnicos o lingüísticos de la persona? |
| 16 | Responsabilidad | Las personas con discapacidad tienen acceso a una evaluación profesional interna e independiente o a un procedimiento de salvaguarda. | ¿La política especifica ante quién, y para qué son responsables los prestadores de servicios? |
| 17 | Prevención |  | ¿La política apoya a las personas con discapacidad en la búsqueda de la prevención primaria, secundaria, y terciaria de las condiciones de salud? |
| 18 | Desarrollo de capacidades |  | ¿La política apoya el desarrollo de la capacidad del personal de salud y del sistema donde trabajan, para abordar las necesidades de salud de las personas con discapacidad? |
| 19 | Acceso | Las personas con discapacidad tienen establecimientos de salud accesibles (es decir, transporte, estructura física de las instalaciones, asequibilidad e información comprensible en formatos adecuados). | ¿La política apoya a las personas con discapacidad en acceso físico, económico y de información a los servicios de salud? |
| 20 | Calidad | Las personas con discapacidad tienen garantizada la calidad de los servicios clínicamente adecuados. | ¿La política apoya la calidad de los servicios para personas con discapacidad poniendo de relieve la necesidad de una práctica basada en la evidencia y profesionalmente calificada? |
| 21 | Eficiencia |  | ¿La política apoya la eficiencia proporcionando una forma estructurada de equiparar los recursos del sistema sanitario con las demandas de servicios para atender las necesidades de salud de las personas con discapacidad? |
| **Note:** Concepts adapted from Amin M, MacLachlan M, Mannan H, El Tayeb S, El Khatim A, Swartz L, et al. EquiFrame: a framework for analysis of the inclusion of human rights and vulnerable groups in health policies. Health Hum Rights. 2011;13:1–20, and Wilbur J, Scherer N, Mactaggart I, Shrestha G, Mahon T, Torondel B, et al. Are Nepal’s water, sanitation and hygiene and menstrual hygiene policies and supporting documents inclusive of disability? A policy analysis. Int J Equity Health. 2021;20:157. | | | |
