# Supplementary material for: Inclusion of people with disabilities in Chilean health policy: a policy analysis
Source: Int J Equity Health. 2024 Aug 29;23:174. doi: 10.1186/s12939-024-02259-4 (PMC11360718; doi:10.1186/s12939-024-02259-4)
Supplement: Supplementary file 2 — Supplementary Material 2 [file 12939_2024_2259_MOESM2_ESM.docx]

| **Additional File 2. Aims of health policy documents included** | | | | |
| --- | --- | --- | --- | --- |
| **Nº** | **Year** | **Title** | **Type** | **Health policy aim** |
| 1 | 2016 | National Policy on Childhood and Adolescence 2015-2025 | Policy | To progressively install an institutional system of rights guarantees for children and adolescents and public policy guidance. |
| 2 | 2017 | National Plan on Dementia | Plan | To address dementias at different levels of healthcare and to reduce its impact on society, as well as to improve the care and quality of life of people living with dementia and their immediate environment. |
| 3 | 2017 | National Plan on Mental Health 2017-2025 | Plan | To contribute to improving people's mental health, through sectoral and intersectoral strategies for the promotion of mental health, prevention of mental disorders, guaranteed mental health care and social inclusion, within the framework of the comprehensive health model with a family and community approach. |
| 4 | 2017 | National Policy on Food and Nutrition | Policy | To provide the framework for the development of food and nutrition regulations, strategies, plans, programmes and projects. |
| 5 | 2018 | National Policy on Sexual and Reproductive Health | Policy | To constitute a national reference framework that defines priorities and guides resources for the implementation of sectoral and intersectoral interventions that contribute to improving the sexual and reproductive health of the population. |
| 6 | 2018 | National Plan on Cancer 2018-2028 | Plan | To reduce the incidence and morbidity and mortality attributable to cancer through strategies and actions that facilitate the promotion, prevention, early diagnosis, treatment, palliative care and follow-up of people, improving the survival of people with cancer, favouring their quality of life and that of their families and communities. |
| 7 | 2021 | National Health Policy to address Gender-Based Violence | Policy | That the plans, programmes, guidelines, norms and benefits of the different levels of the health system design, implement and sustain strategies for the promotion, prevention, care, provision of support services, recovery and comprehensive rehabilitation of survivors, victims and their families, as well as people at risk of suffering gender-based violence. |
| 8 | 2021 | National Health Plan for the Elderly and its Action Plan 2020-2030 | Plan | To improve the functional capacity of the elderly, through a long-term National Integrated Health Plan with Strategic Lines and intervention strategies, thus improving subjective well-being and social participation. |
| 9 | 2021 | National Action Plan on Mental Health 2019-2025 | Plan | To strengthen the implementation and management of the National Mental Health Plan 2017-2025, as well as the government's 2018-2022 proposal for mental health, providing a consensual route, with defined strategies, actions and indicators, which facilitate the monitoring of mental health actions and their financing. |
| 10 | 2022 | National Health Strategy for the 2030 Health Goals | Strategy | To establish the Health Objectives for the Decade 2021-2030, and its consequent National Health Plan, which seeks to ensure health rights, achieve universal coverage and reduce health inequities in the population, and whose goal is to achieve high levels of health for the entire population. |
| 11 | 2022 | National Plan on Non-Communicable Diseases | Plan | To build a ‘Situation Analysis of Non-Communicable Diseases (NCDs)’, which gathers the updated scientific evidence on NCDs at international and national level and exposes the main epidemiological data, as well as the strategies and policies in place to address NCDs. |
| 12 | 2022 | National Plan on Oral Health 2021-2030 | Plan | To improve the oral health status of the population throughout the life course with a focus on health equity. |
| **Note:** The documents were published by the Ministry of Health, except for the National Policy on Childhood and Adolescence, which was published by the Ministry General Secretariat of the Presidency, which led a council of ministers, including the Ministry of Health. | | | | |
