# Supplementary material for: Inclusion of people with disabilities in Chilean health policy: a policy analysis
Source: Int J Equity Health. 2024 Aug 29;23:174. doi: 10.1186/s12939-024-02259-4 (PMC11360718; doi:10.1186/s12939-024-02259-4)
Supplement: Supplementary file 3 — Supplementary Material 3 [file 12939_2024_2259_MOESM3_ESM.docx]

| **Additional File 3. Examples of core concepts scored 3 or 4** | | |
| --- | --- | --- |
| **Nº** | **Concept** | **Reference** |
| **1** | **Non-Discrimination** | **[Score 3, National Plan on Mental Health]** Law 20.584 that Regulates the Rights and Duties of Persons in Relation to Actions Related to their Health Care [...] establishes, among others: The right of every person to "receive health promotion, protection and recovery and rehabilitation actions in a timely manner and without any discrimination, and that the care provided to persons with physical or mental disabilities and those deprived of liberty shall be governed by the rules issued by the Ministry of Health, to ensure that it is timely and of equal quality". |
| **2** | **Individualized Services** | **[Score 4, National Action Plan on Mental Health]** Objective: Improve the autonomy and social inclusion of people with mental disorders or disabilities. Initiative on health network management and coordination: Promotion of support services for people with mental disabilities in the health network. Indicator: Number of mental health specialized facilities that incorporate objectives and actions linked to social inclusion in the Comprehensive Care Plans of users/ Total number of facilities in the health network in the country. Goal: 100% by December 2025. |
| **3** | **Entitlement** | **[Score 4, National Health Plan for the Elderly and its Action Plan]** Objetive: Audit. Intervention strategies: Generation, systematisation and dissemination of information. Initiatives: Monitoring of compliance with the preferential care regulations associated with Law 21.168. Actions: Monitor the implementation of the Law on Preferential Care to the Elderly and Persons with Disabilities. Indicator: Health centres that comply with the preferential care regulations associated with Law 21.168/health centres audited. Goal: 100% as of March of each year. |
| **4** | **Capability-based services** | *None scored 3 or 4.* |
| **5** | **Participation** | *None scored 3 or 4.* |
| **6** | **Coordination of Services** | **[Score 4, National Health Plan for the Elderly and its Action Plan]** Objective: Reduce waiting times for the elderly. Intervention strategies: Management and coordination of the health network. Initiatives: Implementation of local protocols for the implementation of the Regulation associated with the Law on Preferential Care for the Elderly and Persons with Disabilities. Actions: Monitor and oversee the development and implementation of local protocols for the Preferred Care Act Regulation. Indicator: Number of facilities that develop local preferential care protocol for the implementation of the Regulation associated with Law 21.168 in period t/ Health centres supervised by the Superintendence of Health in period t. Goal: 100% as of December each year. |
| **7** | **Protection from Harm** | **[Score 3, National Health Policy to address Gender-Based Violence]** Forced sterilisations: corresponds to the application of sterilisation interventions on a permanent basis and for contraceptive purposes, particularly to children and adolescents in a situation of disability. In this regard, the CEDAW Committee in its Concluding Observations on the Seventh Periodic Report of Chile (2018) recommends the State of Chile to guarantee the full implementation of the national guidelines on fertility regulation (2018) by ensuring that the "informed consent" procedure is requested by medical personnel prior to sterilisation. Practitioners who perform sterilisations without such consent should be sanctioned. Redress and financial compensation should be available to women victims of non-consensual sterilisation (CEDAW/C/CHL/CO/7). |
| **8** | **Liberty** | **[Score 4, National Policy on Childhood and Adolescence]** Generate a specific diagnosis of the various forms of violence that occur in our country through the visualisation, quantification and characterisation of the different forms established by the Committee on the Rights of the Child. Similarly, assess the particular forms of violence to which children and adolescents with disabilities may be subjected, such as forced sterilisation, violence inflicted under the guise of medical treatment and deliberate disability to exploit them for purposes of begging. The assessment should incorporate the existing institutional response with an evaluation of its effectiveness and relevance. |
| **9** | **Autonomy** | **[Score 3, National Plan on Mental Health]** Strategy: Implementing actions that lead to overcoming the model of substitution of the will of the person with a mental disability by a system of support for the effective exercise of their rights. |
| **10** | **Privacy** | *None scored 3 or 4.* |
| **11** | **Integration** | **[Score 4, National Plan on Oral Health]** Objective: Strengthening the evaluation stage in the oral health policy cycle. Initiative: Assess the coverage of oral health programmes considering the social determinants of health. Actions: Hold working meetings with the Ministry of Social Affairs to integrate information systems. Process indicators: Report on coverage of oral health programmes implemented in primary healthcare centres, disaggregated by sex, age and disability status. |
| **12** | **Contribution** | *None scored 3 or 4.* |
| **13** | **Family Resource** | *None scored 3 or 4.* |
| **14** | **Family Support** | **[Score 3, National Plan on Mental Health]** Strategy: Generating programmes and actions to support family members and carers of people with mental disabilities, which have a positive impact on them, on the person being cared for and on their family environment. |
| **15** | **Cultural responsiveness** | **[Score 3, National Plan on Mental Health]** Strategy: Implementing a mass media plan, including a dedicated website and participation in social media, to disseminate relevant information to users, families and the community in relation to mental health, with special emphasis on the rights of people with mental disabilities, and stigma and mental health. |
| **16** | **Accountability** | **[Score 4, National Plan on Mental Health]** Strategic Objective N°3: Maintain current regulatory instruments up to date and develop the necessary regulations to safeguard the rights of people with mental illness, in terms of access to health and social inclusion, incorporating into the legislation considerations regarding human rights and social determinants such as socio-economic level, geographical dispersion, gender, disability, international migrant population and belonging to indigenous peoples, as well as vulnerable populations such as elderly people, children and adolescents at psychosocial risk and in protection systems and people deprived of their liberty. |
| **17** | **Prevention** | **[Score 3, National Plan on Mental Health]** In the next 10 years, the number of schools promoting mental health should be increased, with defined strategies to improve school coexistence spaces, early detection of mental problems or disorders and effective linkage flows with the health care network and other existing offers at the community level. For their part, health teams are expected to have a greater presence in schools to carry out promotional and preventive actions in the field of mental health and to be able to provide a more efficient and timely response for assessment and intervention as appropriate. In addition, schools are expected to implement inclusive, non-discriminatory policies and practices towards all children and adolescents, especially those with physical, sensory, intellectual, mental or other disabilities. |
| **18** | **Capacity building** | **[Score 4, National Action Plan on Mental Health]** Objective: Develop standards and technical orientations. Initiative: To update policy documents in accordance with the purpose, values, principles and Lines of Action of the National Mental Health Plan 2017-2025. Indicator: By the year 2022 there is a regulation for residential care for people with mental disabilities. |
| **19** | **Access** | **[Score 4, National Plan on Oral Health]** Objetive: Assess the coverage of oral health programmes considering the social determinants of health. This initiative considers completing existing health records with variables such as ethnicity, migrant status, disability status and social vulnerability, some of which are available in the National Territorial Information Coordination System (SNIT) of the Ministry of National Assets or in records managed by other ministries. It is proposed to incorporate the geographical distribution and rurality of the beneficiaries, as well as gender, age, ethnicity, migrant status, disability status and social vulnerability in the evaluation of the coverage of oral health programmes. |
| **20** | **Quality** | **[Score 4, National Plan on Mental Health]** Strategy: Strengthen links with non-governmental organisations (NGOs) that provide services in agreement with the health sector to people with mental illness and/or disability, especially in the area of social inclusion, through the generation of quality standards for community services and with a rights-based approach, establishing fees in accordance with the services required, generating joint training plans, with full integration into the thematic network of mental health, implementing systems of accompaniment, supervision and monitoring, among other actions. |
| **21** | **Efficiency** | **[Score 3, National Action Plan on Mental Health]** In order to advance in the plan to close the gap in mental health services, the Action Plan incorporates actions aimed at implementing the Mental Health Network Management Model: [...] implementation of a system of graduated support for people with disabilities resulting from mental illness; progress in the process of deinstitutionalisation of people in psychiatric hospitals and long-stay clinics in partnership with the Health Services; and implementation of a system for evaluating user satisfaction in mental health, among other actions. |
